# Foodborne and Food-Handler Norovirus Outbreaks: A Systematic Review

**DOI:** 10.1089/fpd.2018.2452

**Published:** 2018-10-10

**Authors:** Joanne L. Hardstaff, Helen E. Clough, Vittoria Lutje, K. Marie McIntyre, John P. Harris, Paul Garner, Sarah J. O'Brien

**Affiliations:** ^1^ Department of Psychology, Health and Society, University of Liverpool, Liverpool, United Kingdom.; ^2^ Cochrane Infectious Diseases Group, Liverpool, United Kingdom.; ^3^ Department of Epidemiology and Population Health, Institute of Infection and Global Health, University of Liverpool, Liverpool, United Kingdom.; ^4^ NIHR Health Protection Unit in Gastrointestinal Infections, Liverpool, United Kingdom.; ^5^ Liverpool School of Tropical Medicine, Pembroke Place, Liverpool, United Kingdom.

**Keywords:** foodborne, food handler, norovirus, outbreaks

## Abstract

Norovirus (NoV) is the commonest cause of gastrointestinal disease in the United Kingdom and in many developed countries, causing diarrhea and vomiting in millions of cases worldwide annually. Transmission is most often mediated from person to person. NoV infection has, however, additionally been associated with the consumption of food, either through the consumption of food contaminated at source such as seafood, berries, and salad, or as a consequence of the foodstuff being contaminated in some way by a food handler during processing or serving. A systematic review of outbreaks attributed to NoV between January 2003 and July 2017 was conducted to assess the contribution of food handlers to the burden of NoV, and to identify foods commonly associated with NoV outbreaks. A total of 3021 articles were screened, of which 27 met the definition of confirmed foodborne outbreaks and 47 met the criteria for definite food-handler NoV outbreaks. Of all food types, shellfish were implicated in the greatest number of definite foodborne outbreaks. Food handlers contributed to definite food-handler outbreaks involving a diverse range of foodstuffs and in a wide variety of settings, including weddings and military establishments. More genotypes of NoV were found in people who were ill than in samples from food and food handlers. The potential for both food products and food handlers to contribute to the burden of NoV infection is demonstrated conclusively.

## Background

Norovirus (NoV) is the leading cause of acute gastroenteritis worldwide (Scallan *et al.*, 2011; Tam *et al.*, 2012; Al-Thani *et al.*, 2013) and leads to sudden onset vomiting and diarrhea. Symptoms usually last for 2 to 4 days (Graham *et al.*, 1994; Rockx *et al.*, 2002; Lopman *et al.*, 2004) in healthy adults (Murata *et al.*, 2007). Symptom duration can be longer in hospitalized patients (Lopman *et al.*, 2004; Murata *et al.*, 2007; O'Ryan *et al.*, 2010). Asymptomatic and symptomatic individuals excrete NoV and can transmit it to other people (Akihara *et al.*, 2005; Huynen *et al.*, 2013; Krumkamp *et al.*, 2015). Asymptomatic individuals tend to shed NoV in smaller amounts than symptomatic individuals (Bernstein *et al.*, 2015). Viral shedding can occur for 3–14 h before symptom onset (Atmar *et al.*, 2008). Cases who are immunocompromised, elderly, and newborn infants can shed virus longer than healthy adults (Atmar *et al.*, 2008). Peak shedding is from 2 to 5 days postinfection (Graham *et al.*, 1994; Atmar *et al.*, 2008; Kirby *et al.*, 2014). The virus survives on fomites, for example, in care homes (Wu *et al.*, 2005) and hospitals (Nenonen *et al.*, 2014). NoV has experimentally survived on surfaces for extended periods of time, enabling long periods of potential exposure (Lamhoujeb *et al.*, 2009; Liu *et al.*, 2009).

Infection with NoV occurs through ingestion, either through contact with NoV in the environment or directly from contaminated food or water. Water samples contaminated with NoV have caused illness in subjects up to 2 months postcontamination (Seitz *et al.*, 2011). Sewage-contaminated water supplies containing NoV have been implicated in large outbreaks, for example, in Nokia, Finland (Rasanen *et al.*, 2010). Food is contaminated indirectly, for example, through sewage being discharged in areas where seafood is farmed (Le Guyader *et al.*, 2008) or from contaminated irrigation water (El-Senousy *et al.*, 2013). It may also be contaminated directly through the hands of infected agricultural workers. For example, Leon-Felix *et al.* (2010) demonstrated contamination of peppers in the field and on the hands of pickers, classifiers, and packers. Furthermore, a food handler who returns to work after acute symptoms of NoV infection have subsided but before the infectious period has ended runs the risk of contaminating food products through unsterile practices during preparation (Parashar *et al.*, 1998). The presence of NoV on the hands of food handlers means that it can easily be transferred to and between utensils, work surfaces, and food (Sharps *et al.*, 2012; Stals *et al.*, 2013a; Tuladhar *et al.*, 2013; Verhaelen *et al.*, 2013; Ronnqvist *et al.*, 2014).

The aim of this review was to assess the contribution of particular foods to definitively foodborne outbreaks of NoV, and to describe the contribution of food handlers to NoV outbreaks.

## Methods

### Literature search

The search window was from January 2003 to July 2017. Databases and websites searched were as follows: Web of Science, Medline, Embase, Biosis previews, CABI (CAB Abstracts^®^ and Global Health^®^), Scopus, Biomed Central, ScienceDirect, OpenSIGLE, Proquest Dissertations and Theses A&I, Foodbase website, Public Health England through www.gov.uk, Cefas through the Defra website, and the World Health Organization website.

The databases, dates of use, and number of articles retrieved are appended in Supplementary Table S1 (Supplementary Data are available online at www.liebertpub.com/fpd). The full list of search terms used for each database search is included in Supplementary Data S1 “Search strategies.” For example, the search terms used for Web of Science were as follows: TOPIC: (norovirus or norwalk or winter vomiting or noroviral) and TOPIC: (foodborne or food-borne or orofecal or orofaecal or sewage or irrigation or hand* or hotel or restaurant* or catering or cook* or waiter* or cruise or canteen or contaminat* or aerosol* or spray* or toilet* or latrine* or utensil* or kitchen* or shellfish or fish* or mussel* or oyster* or strawber* or raspberr* or lettuce or salad* or vegetable* or green* or fruit* or ice or blueberr* or onion* or tomato*).

### Study inclusion criteria

All titles and abstracts were screened by at least two reviewers. Articles were assessed for inclusion by one reviewer. A subsample of titles was then selected at random, and the decision to include or exclude each article was cross-validated by two reviewers.

If an article describing a given outbreak provided formal evidence of laboratory confirmation of NoV infection in both human cases and foodstuffs, that outbreak was classed as definitely foodborne.

If a report describing a given outbreak included laboratory confirmation of infection in both patrons and food handlers, with either the same strain being identified in either handlers or cases, or in handlers and in foods consumed by cases, that outbreak was classified as definitively attributed to food handlers. Genotypes of strains found in food handlers and foods were reported where possible.

### Data collection

Data from each article were collated into a single data abstraction sheet (Supplementary Data S2). In brief, for studies of foodborne infection, information was collected about foods implicated and number of samples taken. For food-handler studies, information was collected on settings, foods handled, and, where possible, number and types of samples tested from food handlers and NoV genotypes.

### Analyses

We calculated the proportion of foodstuffs and people in which NoV was detected and described the genogroups and genotypes present, where recorded. The heterogeneity between articles in terms of study design, timeframes, and study populations prohibited a formal statistical meta-analysis; however, descriptive statistics (proportions, medians, and interquartile ranges) were calculated for data extracted from articles that met the inclusion criteria.

## Results

### Literature search

**FIG. 1.**
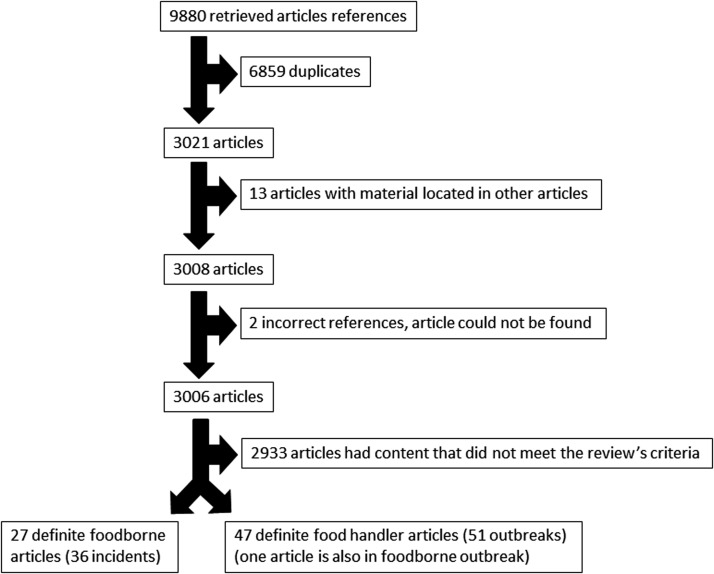
Article selection process.

A total of 9880 articles were originally retrieved from the search strategies (Supplementary Table S1). A total of 6859 article duplicates were removed, leaving 3021 articles to screen. Of these 2933 (97%) did not meet the inclusion criteria and were excluded because they were review articles or they concentrated on diagnostics, artificial contamination, efficacy of decontamination, etiology, and outbreak control; 66 (2.2%) article were duplicates; 13 (0.4%) articles had information found in other articles; and 2 references (0.06%) were incorrect and the articles could not be found (Fig. 1).

Twenty-seven articles met the criteria for inclusion as NoV -definite foodborne outbreaks document 36 separate incidents. The citations are included in Supplementary Data S3.

Outbreaks that met the definition for definite food-handler outbreaks of NoV were identified in 47 articles documenting 51 different outbreaks, the citations can be found in Supplementary Data S4. The Rasmussen 2016 article described an aggregated outbreak report from nine different venues but did not provide any further information that could individualize each outbreak, hence it is included as one aggregate outbreak.

One article had information that was relevant to both foodborne and food-handler outbreaks (Baker *et al.*, 2011) (Fig. 1).

### NoV foodborne outbreaks

NoV foodborne outbreaks were reported from around the globe; however, the largest proportion (57%) was reported in Europe. Of all studies, the most commonly implicated food vehicle in outbreaks was seafood (61%), of which 89% were oysters (Table 1).

**Table 1.** Food Implicated in Foodborne Norovirus Outbreaks

*Country/Food*	*Clams*	*Lettuce*	*Mussels*	*Oysters*	*Raspberries*	*Shellfish*
Australia				1		
Canada				1		
Denmark		1				
Finland					1	
France				3		
Italy			1			
Italy and France				1		
Japan	1					1
New Zealand				2		
Singapore				1		
Sweden				1	1	
United Kingdom				1		
United States				2		
France, Italy				1		
Denmark, France, and United Kingdom				1		

The number of people exposed to NoV in each outbreak ranged from 2 (Muller *et al.*, 2016) to 1580 people (Simmons *et al.*, 2007), with a median of 59 people. The number of people falling ill ranged between 2 (Muller *et al.*, 2016) and 305 (Ng *et al.*, 2005) (median = 23 people). The number of ill people providing samples for testing was between 1 (David *et al.*, 2007; Fitzgerald *et al.*, 2014) and 42 (Le Guyader *et al.*, 2006) (median = 8 people). The median (interquartile range) for the proportion of NoV-positive samples was 79% (52–100%), with the median number of positive samples being 3 (range = 1–24) (Prato *et al.*, 2004; David *et al.*, 2007; Nenonen *et al.*, 2009; Institute of Environmental Science and Research, 2011; Centers for Disease Control and Prevention, 2012; Fitzgerald *et al.*, 2014; Muller *et al.*, 2016).

Most commonly, the NoV genotypes found in food (Table 2) and patrons (Supplementary Table S2) were mixed. Supplementary Tables S2 and S3 indicate that a greater diversity of genotypes was recovered from people affected by the implicated foods than from the foods themselves. The most common genotypes include GII.4 recovered from food and GII.4, GI.4, GI.1, and GI.2 identified from people infected in foodborne outbreaks.

**Table 2.** Settings and Foods Handled in Food Handler-Associated Norovirus Outbreaks

*Food handled/setting*	*Bakery*	*Birthday party*	*Care home*	*Caterer*	*College*	*Healthcare facility*	*Hotel*	*Military base*	*Restaurant*	*School*	*Staff canteen*	*Tourists*	*Wedding*
Aemono sauce									1				
Antipasti and garlic mashed potatoes									1				
Bakery products	1	1								1			1
Cold sausage; meat dish with salad and a rolled spinach pancake						1							
Cold shredded chicken set meal									1				
Egg mayonnaise; sandwiches.							1						
Mushroom dish													1
Paella and beef												1	
Sandwiches							1						
Sandwiches and vegetation						1							
Seafood									3				
Seafood, lamb, and crème brulee									1				
Seafood, passion fruit, and lavender dish									1				
Seafood, spiced chicken, and noodle salad				1									
Standard hospital diet						1							
Unknown			1	1	1	1	3		2	2	1		
Vegetation				1				1		1	3		
Vegetation, water fountain								1					
Vegetation, Sara Udon noodles									1				

Many studies were outbreak reports and had used genotyping and attack rates to determine transmission route. However, 15 of 27 articles included the odds and risk ratios of foods implicated in associated outbreaks (Supplementary Table S3). The risk factors were predominantly seafood related.

### NoV food-handler outbreaks

Food-handler outbreaks occurred in a wide variety of settings (Table 2). The most common setting was restaurants (12/51 outbreaks). In 33% of food-handler outbreaks, implicated food items were not categorized. When they were categorized, the most common, associated with 20% of outbreaks, were salad and vegetables, followed by dishes containing seafood (Supplementary Table S4). The number of patrons with NoV varied from 3 (Sala *et al.*, 2009; Baker *et al.*, 2011; Made *et al.*, 2016) to 660 (Hirakata *et al.*, 2005).

Food handling (mostly kitchen) staff were sampled for NoV in 44 of 51 (86%) outbreaks. The median proportion of positive samples obtained from food handlers was 46% (interquartile range of 25–76%).

The most common genotype recovered from food handlers (Supplementary Table S5) and people whose illness was associated with food handlers (Table 3) was GII.4.

**Table 3.** The Odds and Risk Ratios Calculated in Studies Describing Foodborne Norovirus Outbreaks

*Author*	*Year*	*Risk factor(s)*	*Odds ratio (95% confidence interval)*
Doyle *et al.*	2004	Oysters	55.3 (2.9–1058.7)
Prato *et al.*	2004	Cooked mussels	1.5 (1.05–2.23)
Prato *et al.*	2004	Cooked mussels	3.04 (1.26–7.30)
Prato *et al.*	2004	Raw mussels	1.38 (1–1.91)
Prato *et al.*	2004	Raw musels	1.5 (1.18–1.89)
Ng *et al.*	2005	Oysters	18.3 (9.9–33.2)
Simmons *et al.*	2007	Oysters	11.9 (3.9–36.1)
Simmons *et al.*	2007	Salmon contaminated by oysters	2.3 (1.2–4)
Simmons *et al.*	2007	Tuatuas	3 (1.7–5.6)
Simmons *et al.*	2007	Seafood chowder	2.5 (1–6.3)
Webby et al.	2007	Grilled oysters	17 (5–51)
Webby *et al.*	2007	Oyster cocktails	35 (5–243)
Liko *et al.*	2009	Oysters	11.8 (2–50)
Maunula *et al.*	2009	Frozen raspberries	3
Ethelberg *et al.*	2010	Lettuce	6.2 (1–38)
NZ public health surveillance	2011	Oysters at a wedding	8.5 (2.3–31.3)
Baker *et al.*	2011	Oysters	(11.7-inf)
Baker *et al.*	2011	Lamb	(3.8-inf)
Baker *et al.*	2011	Crème brulee	16.8 (1.3–825.9)
Viriot *et al.*	2011	Late cases—oysters	32.22 (7.09–146.34)
Viriot *et al.*	2011	Early cases—oysters	2.68 (1.36–5.27)
Viriot *et al.*	2011	Early cases—knuckle of ham	3.75 (1.91–7.35)
Muller *et al.*	2016	Salmon and leafy greens	7.7 (2.2–27)
Park *et al.*	2015	Raw seaweed with vinegar and radish	7.9 (1.1–56.2)
Park *et al.*	2015	Seasoned green seaweed with pears	5.1 (1.1–24.8)
Park *et al.*	2015	School A: cabbage kimchi (lunch)	4.56 (2.96–7.02)
Park *et al.*	2015	School A: spicy potato stew (lunch)	1.78 (1.05–3.02)
Park *et al.*	2015	School A: cabbage kimchi (dinner)	1.9 (1.39–2.60)
Park *et al.*	2015	School B: cabbage kimchi	2.26 (1.24–4.15)
Park *et al.*	2015	School C: kimchi	2.10 (1.68–2.63)
Park *et al.*	2015	School C: water	1.56 (1.17–2.08)
Park *et al.*	2015	School C: jajangbap, rice and Chinese bean sauce	3 (1.48–6.09)
Park *et al.*	2015	School C: bean paste soup with tofu	1.23 (1.01–1.50)
Park *et al.*	2015	School C: sweet and sour pork and salad	3.08 (1.59–5.98)
Park *et al.*	2015	School C: tangerine juice	2.55 (1.37–4.74)
Le Guyader *et al.*	2008	Oysters	4.5 (1.6–13.3)

Many food-handler outbreaks had more than one risk factor (Table 4); some focused on a time or place that an exposure occurred, whereas others implicated consumers' dishes that food handlers handled (Table 4).

**Table 4.** The Odds and Risk Ratios Calculated in Studies About Food-Handler Norovirus Outbreaks

*Author*	*Year*	*Risk factor(s)*	*Odds or risk ratio (95% confidence interval)*
Wadl *et al.*	2010	Salad	8.1 (1.5–45.4)
Zomer *et al.*	2010	Eating tomatoes	5.6 (3.2–9.6)
Cai *et al.*	2013	Eating restaurant A	3.46 (1.07–11.16)
Cai *et al.*	2013	Cold shredded chicken set meal	17.82 (4.46–78.17)
Ruan *et al.*	2013	Unknown	12 (5.4–28)
Friedman *et al.*	2005	Wedding cake with strawberry filling	9.3 (6.2–13.8)
Baker *et al.*	2011	Oysters	(11.7-inf)
Baker *et al.*	2011	Lamb	(3.8-inf)
Baker *et al.*	2011	Crème brulee	16.8 (1.3–825.9)
Godoy *et al.*	2016	Eating in canteen	5.8 (1.8–19.3)
Lin *et al.*	2015	Eating a kebab	6.7 (3.4–28)
Sanchez *et al.*	2017	Cake	10.1 (1.2–81.6)
Sanchez *et al.*	2017	Pizza	3.6 (1.1–11.9)
Godoy *et al.*	2005	Sandwiches	2.3 (1.1–5.1)
DeWit *et al.*	2007	Bread rolls	2 (1.6–2.4)
Hirakata *et al.*	2005	Sara Udon	3.1 (1.1–8.7)
Hirakata *et al.*	2005	Spring roll	2.3 (1.1–4.7)
Hirakata*et al.*	2005	Broccoli	2.4 (1.2–4.6)
Centers for Disease Control	2006	Scalloped potatoes	2.8 (1.1–6.9)
Centers for Disease Control	2006	Chicken	2.2 (1.0–4.8)
Centers for Disease Control	2006	Self-reported direct contact ill people	2.3 (1.0–5.0)
Centers for Disease Control	2007	Antipasti platter	2.96 (1.08–8.14)
Centers for Disease Control	2007	Garlic mashed potatoes	4.05 (1.37–11.99)
Schmid *et al.*	2007	Food exposure Wednesday	18.81 (11.82–29.96)
Schmid *et al.*	2007	Food exposure Thursday	2.14 (1.65–2.79)
Schmid *et al.*	2007	Salad	2.82 (1.0–7.94)
Showell *et al.*	2007	Eating salad on day 1	74 (8–1685)
Showell *et al.*	2007	Eating salad on day 2	27 (6–138)
Ohwaki *et al.*	2009	Eating standard diet (workers)	18.13 (5.76–57.03)
Ohwaki *et al.*	2009	Eating standard diet (patients)	2.12 (1.05–4.31)
Nicolay *et al.*	2011	Egg mayonnaise sandwich	2.3 (1.4–3.9)
Nicolay *et al.*	2011	Turkey and stuffing sandwich	1.9 (1.2–3.2)
Nicolay *et al.*	2011	Chicken sandwich	1.9 (1.1–3.1)
Schmid *et al.*	2011	Sliced pork with salad	1.8 (1.1–2.99)
Schmid *et al.*	2011	Rolled pancake filled with spinach	1.86 (1.19–2.93)
Smith *et al.*	2012	Oyster, passion fruit, and lavender dish	7 (1.1–45.2)
Maritschnik *et al.*	2013	Females eating a mushroom dish	2.3 (1.21–4.34)
Ruan *et al.*	2013	Eating delicatessen food from a shop on 14 November	9.7 (2.6–36)
Ruan *et al.*	2013	Eating delicatessen food from a shop on 15 November	8.8 (3.2–24)
Thornley *et al.*	2013	Italian sushi	3.4 (1.2–9.5)
Thornley *et al.*	2013	Consuming food prepared manually	6.6 (2.2–39.2)
Thornley *et al.*	2013	Attending an event before 11.45 a.m.	7.2 (24.–43.2)
Kimura *et al.*	2012	Eating on 23 March	18.1 (9.2–35.4)
Liu *et al.*	2015	Roasted duck	4.94 (2.01–12.35)
Raj *et al.*	2017	Event two: prawn salad	3.92 (1.39–11.08)
Raj *et al.*	2017	Event two: chicken simmered in wine	3.92 (1.39–11.08)
Raj *et al.*	2017	Event three: spring rolls	11.52 (4.31–30.79)
Raj *et al.*	2017	Event six: prawn salad	11.07 (1.33–92.46)
Raj *et al.*	2017	Event six: spicy jelly fish	15.58 (4.41–55.13
Raj *et al.*	2017	Event six: deep fried prawn	5.45 (1.43–20.72)
Smith *et al.*	2017	Ham hock	6.62 (2.19–20.03)
Watier-Grillot *et al.*	2017	Shrimp salad	2.6 (1.2–6.0)
Watier-Grillot *et al.*	2017	Pasta salad	2.9 (1.3–6.4)
Centers for Disease Control	2007	Mashed potatoes	2.4 (1.0–5.4)

## Discussion

### Findings

Oysters and other types of seafood dominated the list of foodstuffs tested for NoV after clinical illness, and it is not clear whether this is a genuine food-related effect, or a consequence of various factors. First, investigator bias may arise because of a long-established association between seafood (Murphy *et al.*, 1979) and NoV. Second, seafood may additionally dominate food sources in our review because of the availability of oysters for testing from batches implicated in outbreaks. This is unlike salad and berries, which are likely to perish or be consumed in their entirety more quickly. Finally, the dominance of seafood may be because of virus particles attaching more easily to seafood than to salad and berries (Tian *et al.*, 2011). Lettuce and raspberries are also implicated in a number of outbreaks, reflecting contamination of food through roots as a result of contaminated irrigation water (Dicaprio *et al.*, 2012; Hirneisen, 2012), contamination by food handlers, or a combination of both.

European law states that food handlers should notify their employers if they are ill and that no toxins should be shed where food is present (European Union 2004). Current advice suggests that food handlers suffering from gastroenteritis should stay away from work for a further 48 h, once the symptoms have disappeared (Food Standards Agency, 2009). Despite this, because of prodromal, prolonged, and asymptomatic shedding, there is the potential for both symptomatic and asymptomatic individuals to contaminate the workplace. In a study by Sabria *et al.* (2016), food handlers and healthcare workers were sampled in workplaces where NoV outbreaks had occurred. In total, 59.1% of workers were found to be excreting NoV and around 70% of those NoV excreters were classed as asymptomatic (Sabria *et al.*, 2016). Sabria *et al.* (2016) also demonstrated that both asymptomatic and symptomatic food workers shed virus for up to 3 weeks postoutbreak exposure (Sabria *et al.*, 2016). Some articles in our review described workers becoming ill at work, resulting in workplace contamination (which could have made it easier to determine the cause of an outbreak) (Centers for Disease Control Prevention, 2007; Baker *et al.*, 2011; Maritschnik *et al.*, 2013; Thornley *et al.*, 2013). Some food handlers, however, were not ill but were found to be shedding the virus (Ozawa *et al.*, 2007) whereas others, who had been around ill people but had not exhibited symptoms themselves, may be asymptomatic shedders who run the same risk as symptomatic individuals of contaminating the workplace (Kuo *et al.*, 2009; Lin *et al.*, 2015).

A study by Verhoef *et al.* (2013) found that few food handlers in catering companies (20%, k = 600, *n* = 1023) had heard of NoV, compared with food handlers based in hospital (92%, k = 141, *n* = 154) and nonhospital (71%, k = 88, *n* = 101) institutions such as nursing homes and retirement homes. Knowledge may impact on a worker's likelihood of staying away from work in the event that they experience an active infection. Fewer facilities necessary for kitchen staff to maintain high standards of hygiene, for example, hand washing instructions for new staff and separate sinks for hand washing were found in catering companies than in hospital restaurants and nonhospital institutional catering (Verhoef *et al.*, 2013). The differences in kitchen standards, training, and knowledge may help to explain why fewer food-handler outbreaks were attributed to hospitals than to restaurants and caterers.

Hedberg *et al.* (2006) found that in restaurants with managers who had undertaken training on food safety, outbreaks were less likely than in those without trained managers and staff. However, practices that reduced contamination such as using gloves and designated utensils on different products did not always occur, even if workers were aware that they should be doing this (Robertson *et al.*, 2013). Hedberg *et al.* (2006) additionally found that outbreaks were less likely in restaurants where sick pay was provided and a staff reporting policy in the event of illness was in place. This is in accordance with a study by Carpenter *et al.* (2013), which found that people continued to work through diarrhea and vomiting for fear of losing their jobs and shifts if they were absent. It is difficult to make recommendations to stay at home in a culture in which many workers will not have regulated hours, and will not necessarily receive sick pay if they are absent from work.

### Limitations

There are various limitations in the results of our review. For example, varying time lags between falling ill and fecal sampling in different studies were observed and this will have affected the likelihood of finding virus. This is noted in two studies as a possible explanation for heterogeneity in shedding periods (Murata *et al.*, 2007; Atmar *et al.*, 2008). The length of time from acquisition of the virus to genotyping may determine the strains found and will not necessarily capture chance-point mutations or gene transfer from other cocirculating strains. This might have resulted in identifying fewer food-handler outbreaks. Furthermore, food handlers will not necessarily admit to being ill (Carpenter *et al.*, 2013; Verhoef *et al.*, 2013), as they may lose work and may not want to leave the workplace understaffed. This will also lead to an underestimate of the frequency of food-handler outbreaks.

Further limitations include the fact that detection of NoV in food and environmental samples is not necessarily widely employed outside specialist laboratories at present (Stals *et al.*, 2013b). There are standardized valid laboratory protocols for examination of hepatitis A virus and NoV in foods, but these are currently qualitative in nature (although ISO/TS 15216-2:2013 is being reviewed and will be replaced by a quantitative standard (ISO/DIS 15216-2)) (Stals *et al.*, 2013b; Anonymous, 2017) and there are challenges in assessing whether or not the NoV detected in human or food samples has infectious potential (Knight *et al.*, 2013).

The completeness of studies included in a systematic review was achieved through the use of a comprehensive search strategy from a wide range of sources. However, the timescale of the review was restricted to ensure comparability of laboratory methods, which will have resulted in the omission of studies before 2003.

Peer-reviewed publication usually requires reporting of novel findings (new virus type, new food vehicle, etc.) and so outbreaks that provide high-quality evidence of long-established causes and exposure routes may not reach the peer-reviewed literature. This means that the burden of illness associated with particular food sources and risky environments may be underrepresented in our systematic review.

The strict case definition resulted in comparatively few articles for which the quality of evidence confirming a food source or food-handler involvement was judged to be high. Relatively few studies had tested both cases and foods, or cases and handlers.

Finally, studies from wealthier countries comprised the majority of those appearing in the review, reflecting the greater technological development, public health infrastructure, and monetary resource required for the investigation of outbreaks and identification of causative microbiological agents. Furthermore, a short duration of illness with NoV may limit the number of outbreaks that are formally reported and investigated, for example, small foodborne outbreaks may be expected and, therefore, not reported in countries in which a lot of seafood, including oysters, is eaten, for example, Japan (*Pers. Comm.* Dr Yamanaka).

## Conclusions

Food and food handlers both contribute to outbreaks of NoV. Some outbreaks were attributed to asymptomatic food handlers. Contaminated shellfish were implicated in the greatest number of definite foodborne outbreaks. Food handlers contributed to definite food-handler outbreaks involving a diverse range of foodstuffs and in a wide variety of settings, including weddings and military establishments. More genotypes of NoV were found in ill people than in samples from food and food handlers. The potential for both food products and food handlers to contribute to the burden of NoV infection was demonstrated conclusively.

## Supplementary Material

Supplemental data
