# Supplementary material for: Foodborne and Food-Handler Norovirus Outbreaks: A Systematic Review
Source: Foodborne Pathog Dis. 2018 Oct 10;15(10):589–97. doi: 10.1089/fpd.2018.2452 (PMC6201779; doi:10.1089/fpd.2018.2452)
Supplement: Supplemental data [file Supp_Data.pdf]

Supplementary Data

Supplementary Data S1: Search Strategies for Electronic Databases

OVID MEDLINE(R) In-Process and Other Non-Indexed Citations and Ovid MEDLINE(R) <1946 to present>
Search terms:

1. norwalk virus.mp. or norwalk virus/
2. norovirus.mp. or exp norovirus/
3. small round structured virus.mp.
4. (norovirus or noroviral).ab. or (norovirus or noroviral).ti.
5. (winter and vomiting).ab. or (winter and vomiting).ti.
6. 1 or 2 or 3 or 4 or 5
7. limit 6 to yr = "2003–present"
8. Foodborne diseases/
9. Food contamination/
10. (foodborne or food-borne).mp.
11. (faecal-oral or oro-fecal or oro-faecal).ab. or (faecal-oral or oro-fecal or oro-faecal).ti.
12. (sewage or irrigation or hand* or hotel or restaurant* or catering or cook* or waiter*).ab. or (sewage or irrigation or hand* or hotel or restaurant* or catering or cook* or waiter*).ti.
13. fomites.mp. or Fomites/
14. (contaminat* or aerosol* or spray* or toilet* or latrine* or utensil* or kitchen*).ab. or (contaminat* or aerosol* or spray* or toilet* or latrine* or utensil* or kitchen*).ti.
15. Shellfish poisoning/or shellfish/or shellfish.mp.
16. (fish* or mussel* or oyster* or strawber* or raspberr* or lettuce or salad* or vegetable* or green* or fruit* or ice or blueberr* or onion* or tomato*).ab. or (fish* or mussel* or oyster* or strawber* or raspberr* or lettuce or salad* or vegetable* or green* or fruit* or ice or blueberr* or onion* or tomato*).ti.
17. 8 or 9 or 10 or 11 or 12 or 13 or 14 or 15 or 16
18. 6 and 17

EMBASE OVID 1947 to present, updated daily
Search terms:

1. norwalk virus.mp. or norwalk virus/
2. norovirus.mp. or exp norovirus/
3. small round structured virus.mp.
4. (norovirus or noroviral).ab. or (norovirus or noroviral).ti.
5. (winter and vomiting).ab. or (winter and vomiting).ti.
6. 1 or 2 or 3 or 4 or 5
7. limit 6 to yr = "2003–present"
8. Foodborne diseases/
9. Food contamination/
10. (foodborne or food-borne).mp.
11. (faecal-oral or oro-fecal or oro-faecal).ab. or (faecal-oral or oro-fecal or oro-faecal).ti.
12. (sewage or irrigation or hand* or hotel or restaurant* or catering or cook* or waiter*).ab. or (sewage or irrigation or hand* or hotel or restaurant* or catering or cook* or waiter*).ti.
13. fomites.mp. or fomites/
14. (contaminat* or aerosol* or spray* or toilet* or latrine* or utensil* or kitchen*).ab. or (contaminat* or aerosol* or spray* or toilet* or latrine* or utensil* or kitchen*).ti.
15. Shellfish poisoning/or shellfish/or shellfish.mp.
16. (fish* or mussel* or oyster* or strawber* or raspberr* or lettuce or salad* or vegetable* or green* or fruit* or ice or blueberr* or onion* or tomato*).ab. or (fish* or mussel* or oyster* or strawber* or raspberr* or lettuce or salad* or vegetable* or green* or fruit* or ice or blueberr* or onion* or tomato*).ti.
17. 8 or 9 or 10 or 11 or 12 or 13 or 14 or 15 or 16
18. 6 and 17

Biosis previews

TOPIC: (norovirus or norwalk or winter vomiting or noroviral) and TOPIC: (foodborne or food-borne or orofecal or orofaecal or sewage or irrigation or hand* or hotel or restaurant* or catering or cook* or waiter* or cruise or canteen)

CABI (Abstracts® and Global Health®)

TOPIC: (norovirus or norwalk or winter vomiting or noroviral) and TOPIC: (foodborne or food-borne or orofecal or orofaecal or sewage or irrigation or hand* or hotel or restaurant* or catering or cook* or waiter* or cruise or canteen)

Scopus

History Search Terms:
(TITLE-ABS-KEY [foodborne or food-borne or orofecal or orofaecal or sewage or irrigation or hand* or hotel or restaurant* or catering or cook* or waiter* or cruise or canteen or contaminat* or aerosol* or spray* or toilet* or latrine* or utensil* or kitchen* or shellfish] and SUBJAREA [mult or agri or bioc or immu or neur or phar or mult or medi or nurs or vete or dent or heal]) or (TITLE-ABS-KEY [fish* or mussel* or oyster* or strawber* or raspberr* or lettuce or salad* or vegetable* or green* OR fruit* OR ice or blueberr* or onion* or tomato*] and SUBJAREA [mult or agri or bioc or immu or neur or phar or mult or medi or nurs or vete or dent or heal]) and (TITLE-ABS-KEY [norovirus or norwalk or winter vomiting] and SUBJAREA [mult or agri or bioc or immu or neur or phar or mult or medi or nurs or vete or dent or heal])

Supplementary Data S2: Data Collected

Data were collected for foodborne outbreaks using the following categories: name (author), year, journal, title, country, region, date of outbreak, duration of the outbreak, problems accessing the article, who carried out the investigation, how the virus was detected, case definition for both primary and secondary cases, suspected cause, type of study,

period of recall, did the study include food handlers, demographics, setting, age, gender ratio, number of people at the event (exposed), number of people at the event contacted, number of people responded, number of cases, number of primary cases, number of secondary cases, number of samples, number of samples positive, single or multiple genotypes, genotypes from cases, genotypes from food, other pathogens in cases, number of cases, number of controls, were there matching criteria, number of cases exposed, number of cases unexposed, controls exposed, number of controls unexposed, confounding factors explored, odds ratios and their confidence intervals, population attributable risks and their confidence intervals and risk ratios and their confidence intervals.

Additional data were collected for food handler-associated outbreaks that included the following: number of kitchen handlers, number of ill kitchen handlers, number of kitchen handlers sampled, number of kitchen handlers sampled that were ill, single of multiple strains from kitchen handlers, genotypes from kitchen handlers, other pathogens found in kitchen handlers, and foods handled by kitchen handlers.

Supplementary Data S3: Foodborne Outbreak Articles

(Furuta *et al.*, 2003; Doyle *et al.*, 2004; Le Guyader *et al.*, 2004, 2006, 2008, 2010; Prato *et al.*, 2004; Ng *et al.*, 2005; David *et al.*, 2007; Simmons *et al.*, 2007; Webby *et al.*, 2007; Liko and Keene, 2009; Maunula *et al.*, 2009; Nenonen *et al.*, 2009; Ethelberg *et al.*, 2010; Iizuka *et al.*, 2010; Westrell *et al.*, 2010; Baker *et al.*, 2011; Institute of Environmental Science and Research, 2011b; Viriot *et al.*, 2011; Prevention, 2012; Fitzgerald *et al.*, 2014; Park *et al.*, 2015a, 2015b; Cho *et al.*, 2016; Muller *et al.*, 2016; Rasmussen *et al.*, 2016)

Supplementary Data S4: Food-Handler Outbreak Articles

(Tashima and Chijiwa, 2003; Friedman *et al.*, 2005; Furuya *et al.*, 2005; Godoy *et al.*, 2005, 2016; Hirakata *et al.*, 2005; Kim *et al.*, 2005; Lederer *et al.*, 2005; Sakon *et al.*, 2005; Sala *et al.*, 2005, 2009; Centers for Disease Control Prevention, 2006, 2007; de Wit *et al.*, 2007; Schmid *et al.*, 2007, 2011; Showell *et al.*, 2007; Oogane *et al.*, 2008; Boxman *et al.*, 2009; Medici *et al.*, 2009; Ohwaki *et al.*, 2009; Barrabeig *et al.*, 2010; Wadl *et al.*, 2010; Yu *et al.*, 2010; Zomer *et al.*, 2010; Baker *et al.*, 2011; Institute of Environmental Science and Research, 2011a; Mayet *et al.*, 2011; Nicolay *et al.*, 2011; Kimura *et al.*, 2012; Smith *et al.*, 2012, 2017; Cai *et al.*, 2013; Huang *et al.*, 2013; Maritschnik *et al.*, 2013; Ohe, 2013; Ruan *et al.*, 2013; Thornley *et al.*, 2013; Jung *et al.*, 2015; Lin *et al.*, 2015; Liu *et al.*, 2015; Leshem *et al.*, 2016; Made *et al.*, 2016; Rasmussen *et al.*, 2016; Raj *et al.*, 2017; Sanchez *et al.*, 2017; Watier-Grillot *et al.*, 2017)

Supplementary References

Baker K, Morris J, McCarthy N, *et al.* An outbreak of norovirus infection linked to oyster consumption at a UK restaurant, February 2010. J Public Health (Oxf) 2011;33:205–211.

Barrabeig I, Rovira A, Buesa J, *et al.* Foodborne norovirus outbreak: The role of an asymptomatic food handler. BMC Infect Dis 2010;10:269.

Boxman I, Dijkman R, Verhoef L, *et al.* Norovirus on swabs taken from hands illustrate route of transmission: A case study. J Food Prot 2009;72:1753–1755.

Cai W-F, Xie H-P, Liu Y-F, *et al.* [An epidemiological investigation on a food-born outbreak of noroviru caused by Sydney 2012 G II.4 strain]. Chung Hua Liu Hsing Ping Hsueh Tsa Chih 2013;34:804–807.

Centers for Disease Control Prevention. Multisite outbreak of norovirus associated with a franchise restaurant—Kent County, Michigan, May 2005. MMWR Morb Mortal Wkly Rep 2006;55:395–397.

Centers for Disease Control Prevention. Norovirus outbreak associated with ill food-service workers—Michigan, January-February 2006. MMWR Morb Mortal Wkly Rep 2007;56: 1212–1216.

Cho HG, Lee SG, Lee MY, *et al.* An outbreak of norovirus infection associated with fermented oyster consumption in South Korea, 2013. Epidemiol Infect 2016;144:2759–2764.

David ST, McIntyre L, MacDougall L, *et al.* An outbreak of norovirus caused by consumption of oysters from geographically dispersed harvest sites, British Columbia, Canada, 2004. Foodborne Pathog Dis 2007;4:349–358.

de Wit MAS, Widdowson MA, Vennema H, de Bruin E, Fernandes T, Koopmans M. Large outbreak of norovirus: The baker who should have known better. J Infect 2007;55:188–193.

Doyle A, Barataud D, Gallay A, *et al.* Norovirus foodborne outbreaks associated with the consumption of oysters from the Etang de Thau, France, December 2002. Euro Surveill 2004;9:24–26.

Ethelberg S, Lisby M, Bottiger B, *et al.* Outbreaks of gastroenteritis linked to lettuce, Denmark, January 2010. Euro Surveill 2010;15:11.

Fitzgerald TL, Merritt TD, Zammit A, *et al.* An outbreak of norovirus genogroup II associated with New South Wales oysters. Commun Dis Intell Q Rep 2014;38:E9–E15.

Friedman DS, Heisey-Grove D, Argyros F, *et al.* An outbreak of norovirus gastroenteritis associated with wedding cakes. Epidemiol Infect 2005;133:1057–1063.

Furuta T, Akiyama M, Kato Y, Nishio O. [A food poisoning outbreak caused by purple Washington clam contaminated with norovirus (Norwalk-like virus) and hepatitis A virus]. Kansenshogaku Zasshi 2003;77:89–94.

Furuya Y, Katayama T, Takahashi T, Nikkawa T. Norovirus gastroenteritis in Kanagawa Prefecture in December 2004. Jpn J Infect Dis 2005;58:391–392.

Godoy P, Alseda M, Bartolome R, *et al.* Norovirus gastroenteritis outbreak transmitted by food and vomit in a high school. Epidemiol Infect 2016;144:1951–1958.

Godoy P, Izcara J, Bartolome R, *et al.* Outbreak of food-borne Norovirus associated with the consumption of sandwiches. Med Clin (Barc) 2005;124:161–164.

Hirakata Y, Arisawa K, Nishio O, Nakagomi O. Multiprefectural spread of gastroenteritis outbreaks attributable to a single genogroup II norovirus strain from a tourist restaurant in Nagasaki, Japan. J Clin Microbiol 2005;43:1093–1098.

Huang J, Xu X, Weng Q, *et al.* Serial foodborne norovirus outbreaks associated with multiple genotypes. PLoS One 2013;8:e63327.

Iizuka S, Oka T, Tabara K, *et al.* Detection of sapoviruses and noroviruses in an outbreak of gastroenteritis linked genetically to shellfish. J Med Virol 2010;82:1247–1254.

Institute of Environmental Science and Research. Outbreak case reports. N Z Public Health Surveill Rep 2011a;9:6.

Institute of Environmental Science and Research. Outbreak case reports: Norovirus outbreak linked to consumption of im-

Supplementary Table S1. Databases, Dates of Their Use, and Number of Articles Retrieved for a Systematic Review of Foodborne and Food Handler-Associated Norovirus Outbreaks

Search No.	*Date*	*Database searched*	*Hits (before duplicate removal)*[a]
1	28/07/2017	Medline (OVID)	1920
2	28/07/2017	Embase (OVID)	2265
3	28/07/2017	Web of Science (Science Citation Index Expanded (SCI-EXPANDED)	2229
4	28/07/2017	Biosis previews	1368
5	28/07/2017	CABI (CAB Abstracts® and Global Health®)	862
6	28/07/2017	Scopus	835
7	28/07/2017	Biomed Central	74
8	28/07/2017	ScienceDirect	121
9	28/07/2017	OpenSIGLE	35
10	28/07/2017	Proquest Dissertations and Theses A&I	65
11	28/07/2017	Foodbase website	68
12	28/07/2017	PHE (www.gov.uk)	1
13	28/07/2017	WHO website	18
14	28/07/2017	CEFAS (Defra.gov.uk)	19

[a]The number of articles originally retrieved via the search terms prior to removing duplicate articles.

ported raw oysters. N Z Public Health Surveill Rep 2011b;9:7–8.

Jung S, Hwang B-M, Jeong HJ, *et al.* Occurrence of Norovirus GII.4 Sydney Variant-related Outbreaks in Korea. Osong Public Health Res Perspect 2015;6:322–326.

Kim S-H, Cheon D-S, Kim J-H, *et al.* Outbreaks of gastroenteritis that occurred during school excursions in Korea were associated with several waterborne strains of norovirus. J Clin Microbiol 2005;43:4836–4839.

Kimura H, Nitami K, Mizuguchi A. [Descriptive epidemiology for two outbreak cases of norovirus gastroenteritis in an elementary school]. Nippon Koshu Eisei Zasshi 2012;59:101–111.

Le Guyader FS, Bon F, DeMedici D, *et al.* Detection of multiple noroviruses associated with an international gastroenteritis outbreak linked to oyster consumption. J Clin Microbiol 2006;44:3878–3882.

Le Guyader FS, Krol J, Ambert-Balay K, *et al.* Comprehensive analysis of a norovirus-associated gastroenteritis outbreak, from the environment to the consumer. J Clin Microbiol 2010;48:915–920.

Le Guyader FS, Le Saux J-C, Ambert-Balay K, *et al.* Aichi virus, norovirus, astrovirus, enterovirus, and rotavirus involved in clinical cases from a French oyster-related gastroenteritis outbreak. J Clin Microbiol 2008;46:4011–4017.

Le Guyader FS, Mittelholzer C, Haugarreau L, *et al.* Detection of noroviruses in raspberries associated with a gastroenteritis outbreak. Int J Food Microbiol 2004;97:179–186.

Lederer I, Schmid D, Pichler A-M, *et al.* Outbreak of norovirus infections associated with consuming food from a catering company, Austria, September 2005. Euro Surveill 2005;10: E051020.7.

Leshem E, Gastanaduy PA, Trivedi T, *et al.* Norovirus in a United States virgin islands resort: Outbreak investigation, response, and costs. J Travel Med 2016;23.

Liko J, Keene WE. Use of templates to identify source of norovirus outbreak. Emerg Infect Dis 2009;15:839–840.

Lin Y-C, Hipfl E, Lederer I, Allerberger F, Schmid D. A norovirus GII.P21 outbreak in a boarding school, Austria 2014. Int J Infect Dis 2015;37:25–29.

Liu Y, Tam YH, Yuan J, *et al.* A Foodborne Outbreak of Gastroenteritis Caused by Vibrio parahaemolyticus and Norovirus through Non-Seafood Vehicle. PLoS One 2015;10:e0137848.

Made D, Irmscher HM, Helmecke C, *et al.* Norovirus outbreak in a restaurant: Investigation of the path of infection by sequence analysis of food and human samples. J Consum Prot Food Saf 2016;11:345–351.

Maritschnik S, Kanitz EE, Simons E, *et al.* A Food Handler-Associated, Foodborne Norovirus GII.4 Sydney 2012-Outbreak Following a Wedding Dinner, Austria, October 2012. Food Environ Virol 2013;5:220–225.

Maunula L, Roivainen M, Keranen M, *et al.* Detection of human norovirus from frozen raspberries in a cluster of gastroenteritis outbreaks. Euro Surveill 2009;14.

Mayet A, Andreo V, Bedubourg G, *et al.* Food-borne outbreak of norovirus infection in a French military parachuting unit, April 2011. Euro Surveill 2011;16: pii: 19930.

Medici MC, Morelli A, Arcangeletti MC, *et al.* An outbreak of norovirus infection in an Italian residential-care facility for the elderly. Clin Microbiol Infect 2009;15:97–100.

Muller L, Rasmussen LD, Jensen T, *et al.* Series of Norovirus Outbreaks Caused by Consumption of Green Coral Lettuce, Denmark, April 2016. PLoS Curr 2016;8.

Nenonen NP, Hannoun C, Olsson MB, Bergstrom T. Molecular analysis of an oyster-related norovirus outbreak. J Clin Virol 2009;45:105–108.

Ng TL, Chan PP, Phua TH, *et al.* Oyster-associated outbreaks of Norovirus gastroenteritis in Singapore. J Infect 2005;51: 413–418.

Nicolay N, McDermott R, Kelly M, *et al.* Potential role of asymptomatic kitchen food handlers during a food-borne outbreak of norovirus infection, Dublin, Ireland, March 2009. Euro Surveill 2011;16: pii: 19931.

Ohe M. A ''blind spot'' regarding the norovirus infection pathway. Tohoku J Exp Med 2013;229:125–128.

Ohwaki K, Nagashima H, Aoki M, Aoki H, Yano E. A foodborne norovirus outbreak at a hospital and an attached long-term care facility. Jpn J Infect Dis 2009;62: 450–454.

Oogane T, Hirata A, Funatogawa K, Kobayashi K, Sato T, Kimura H. Food poisoning outbreak caused by norovirus GII/4 in school lunch, Tochigi prefecture, Japan. Jpn J Infect Dis 2008;61:423–424.

Park J-H, Jung S, Shin J, Lee JS, Joo IS, Lee D-Y. Three gastroenteritis outbreaks in South Korea caused by the con-

Supplementary Table S2. Genotypes from Food

Quarter	*Year*	*GI- NLV/Steinbach/ EG/2001/CA; GII-NLV/Tarrag/ 238/2001/Sp, Saitama U25 and Khs1-1997-JP.*	*GI.1*	*GI.1; GI.2; GI.4; GII.4*	*GI.1; GII.3*	*GI.1; GII.4; GII.8*	*GI.2*	*GI.2; GII.17*	*GI.3; GII.3; GII.6; GII.8; GII.13*	*GI.4*	*GI.4; GII.4*	*GII.2*	*GII.3*	*GII.4*	*GII.4; GII.14*	*NA*
Q4	2001															2
Q1/2	2002	1														
Q4	2002					2										
Q4 2002 and Q1 2003											1					
Q4	2003													2		
Q4 2003 and Q1 2004																
Q1	2004						2							1		
Q1	2006			1												
Q2	2006								1							
Q4	2006															1
Q1	2007				1											
Q1	2008												1			
Q2	2008		1													
Q4	2009									1						
Q1	2010															6
Q3	2010															1
Q4	2011												1			
Q1	2012											2				
Q4	2012															1
Q2	2013										3				1	
Q1	2016							1								
Q2	2016															1

Supplementary Table S3. Genotypes People from Foodborne Outbreaks

Quarter	*Year*	*G1- NLV/ Steinbach/ EG/2001/CA; G2 - NLV/ Tarrag/ 238/2001/Sp, Saitama U25 and Khs1-1997-JP*	*G1.4*	*GI.1*	*GI.1; GI.2; GI.9; GI.12; GI.14; GII.6*	*GI.1; GI.2; GII.2; GII.4; GII.7; GII.17; GIIb*	*GI.1; GII.17*	*GI.2*	*GI.2; GI.4; GII.3; GII.6; GII.7; GII.9*	*GI.4*	*GI.4; GI.6; GII.4; GII.8*	*GI.4; GII.4*	*GI.4; GII.4; GII.b*	*GI.P2; GI.2; GI.Pb-GI.6*	*GII.2; GII.4;*	*GII.3; GII.6; GII.12*	*GII.4*	*GII.4, GII.11, GII.14*	*GII.5, GII.6, GII.7, GII.14 amd GII.17*	*GII.P17; GII.17*	*GII; GII.4; GII.5*	*NA*
Q4	2001																					2
Q1/2	2002	1																				
Q4	2002										1		1									
Q4 2002 and Q1 2003												1										
Q4	2003								1													1
Q4 2003 and Q1 2004																						1
Q1	2004							1									1				1	
Q1	2006					1																
Q2	2006															1						
Q4	2006																					1
Q1	2007			1																		
Q1	2008														1							
Q2	2008			1																		
Q4	2009		1																			
Q1	2010																					6
Q3	2010																					1
Q4	2011						1															
Q1	2012				1														1			
Q4	2012																1					
Q2	2013									3								1				
Q1	2016																			1		
Q2	2016													1								

Supplementary Table S4. Food Handlers' Genotypes

Quarter	*Year*	*GI.12*	*GI.2*	*GI.3*	*GI.4*	*GI.6*	*GI.6; GII.4*	*GII.1*	*GII.12.g*	*GII.17*	*GII.2*	*GII.2; GII.4, GII.6*	*GII.3*	*GII.4*	*GII.7*	*GII.9; GII.13*	*GII.P21*	*GIIe; GII.4*	*NA*
Q2	1999							1											
Q1	2001																		1
Q2	2002			1															2
Q4	2003												1						
Q2	2004			1			1												
Q3	2004																		1
Q4	2004	1																	4
Q2	2005																		1
Q3	2005										1								1
Q1	2006				1										1				
Q4	2006													1					
Q1	2007													1					
Q2	2007					1								1					
Q4	2007			1										1					
Q4 2008 and Q1 2009														1					
Q1	2009											1		2		1			
Q4	2009													1					1
Q1	2010								1										1
Q3	2010													1	1			1	
Q2	2011																		1
Q4	2011													3	1				
Q1 and Q2 2012				1															
Q2	2012													1					
Q4	2012													1					
Q4 2012 and Q1 2013														1					
Q1	2013													1					
Q2	2013																		1
Q1	2014																1		
Q3	2014		1																
Q3	2015					1													
Q1	2016									1									1

Supplementary Table S5. Patrons Ill by Food Handlers' Genotypes

Quarter	*Year*	*GI.12; GII.14*	*GI.2*	*GI.3*	*GI.3; GI.4; GI.5, GII.4; GII.6; GII.12; GII.14*	*GI.3; GI.4; GII.5; GII.8*	*GI.4*	*GI.6*	*GI; GII.3; GII.4; GII.6*	*GII.1*	*GII.12.g*	*GII.17*	*GII.2*	*GII.3*	*GII.4*	*GII.7*	*GII.9*	*GII.e; GII.pe/ GII.2*	*GII.P21*	*GIIe; GII.4*	*NA*
Q2	1999									1											
Q1	2001																				1
Q2	2002			1																	2
Q4	2003													1							
Q2	2004			1		1															
Q3	2004																				1
Q4	2004	1																			4
Q2	2005																				1
Q3	2005												1								1
Q1	2006						1									1					
Q4	2006														1						
Q1	2007														1						
Q2	2007							1							1						
Q4	2007			1											1						
Q4 2008 and Q1 2009															1						
Q1	2009								1						2		1				
Q4	2009														1						1
Q1	2010										1										1
Q3	2010														1	1				1	
Q2	2011																				1
Q4	2011														3	1					
Q1 and Q2 2012					1																
Q2	2012														1						
Q4	2012														1						
Q4 2012 and Q1 2013															1						
Q1	2013														1						
Q2	2013																				1
Q1	2014																		1		
Q3	2014		1																		
Q3	2015							1													
Q1	2016											1						1			

sumption of kimchi tainted by norovirus GI.4. Foodborne Pathog Dis 2015a;12:221–227.

Park JH, Jeong HS, Lee JS, *et al.* First norovirus outbreaks associated with consumption of green seaweed (Enteromorpha spp.) in South Korea. Epidemiol Infect 2015b;143:515–521.

Prato R, Lopalco PL, Chironna M, Barbuti G, Germinario C, Quarto M. Norovirus gastroenteritis general outbreak associated with raw shellfish consumption in south Italy. BMC Infect Dis 2004;4:37.

Prevention CfDCa. Norovirus Infections Associated with Frozen Raw Oysters-Washington, 2011. Clin Infect Dis 2012;54:I-I.

Raj P, Tay J, Ang LW, *et al.* A large common-source outbreak of norovirus gastroenteritis in a hotel in Singapore, 2012. Epidemiol Infect 2017;145:535–544.

Rasmussen LD, Schultz AC, Uhrbrand K, Jensen T, Fischer TK. Molecular Evidence of Oysters as Vehicle of Norovirus GII.P17-GII.17. Emerg Infect Dis 2016;22:2024–2025.

Ruan F, Tan AJ, Man TF, *et al.* Gastroenteritis outbreaks caused by Norovirus genotype II.7 in a college in China (Zhuhai, Guangdong) in 2011. Foodborne Pathog Dis 2013;10:856–860.

Sakon N, Yamazaki K, Yoda T, Kanki M, Otake T, Tsukamoto T. A Norovirus outbreak of gastroenteritis linked to packed lunches. Jpn J Infect Dis 2005;58:253.

Sala MR, Arias C, Dominguez A, Bartolome R, Muntada JM. Foodborne outbreak of gastroenteritis due to Norovirus and Vibrio parahaemolyticus. Epidemiol Infect 2009;137:626–629.

Sala MR, Cardenosa N, Arias C, *et al.* An outbreak of food poisoning due to a genogroup I norovirus. Epidemiol Infect 2005;133:187–191.

Sanchez M-A, Corcostegui S-P, De Broucker C-A, *et al.* Norovirus GII.17 Outbreak Linked to an Infected Post-Symptomatic Food Worker in a French Military Unit Located in France. Food Environ Virol 2017;9:234–237.

Schmid D, Kuo HW, Hell M, *et al.* Foodborne gastroenteritis outbreak in an Austrian healthcare facility caused by asymptomatic, norovirus-excreting kitchen staff. J Hosp Infect 2011;77:237–241.

Schmid D, Stuger HP, Lederer I, *et al.* A foodborne norovirus outbreak due to manually prepared salad, Austria 2006. Infection 2007;35:232–239.

Showell D, Sundkvist T, Reacher M, Gray J. Norovirus outbreak associated with canteen salad in Suffolk, United Kingdom. Euro Surveill 2007;12:E071129.6.

Simmons G, Garbutt C, Hewitt J, Greening G. A New Zealand outbreak of norovirus gastroenteritis linked to the consumption of imported raw Korean oysters. N Z Med J 2007;120:U2773.

Smith AJ, McCarthy N, Saldana L, *et al.* A large foodborne outbreak of norovirus in diners at a restaurant in England between January and February 2009. Epidemiol Infect 2012;140:1695–1701.

Smith KC, Inns T, Decraene V, Fox A, Allen DJ, Shah A. An outbreak of norovirus GI-6 infection following a wedding in North West England. Epidemiol Infect 2017;145:1239–1245.

Tashima S, Chijiwa K. An outbreak of SRSV-gastroenteritis among schoolchildren and teachers in early summer. [Japanese]. [Nippon koshu eisei zasshi] Jpn J Public Health 2003;50:225–233.

Thornley CN, Hewitt J, Perumal L, *et al.* Multiple outbreaks of a novel norovirus GII.4 linked to an infected post-symptomatic food handler. Epidemiol Infect 2013;141:1585–1597.

Viriot D, Cochet A, Watrin M, Benoit P, Moyano MB, Golliot F. Investigation of a collective polymicrobial foodborne outbreak, Yves du Manoir Stadium, Montpellier (France), February 2010. Bull Epidemiol Hebd 2011:289–292.

Wadl M, Scherer K, Nielsen S, *et al.* Food-borne norovirus-outbreak at a military base, Germany, 2009. BMC Infect Dis 2010;10:30.

Watier-Grillot S, Boni M, Tong C, *et al.* Challenging Investigation of a Norovirus Foodborne Disease Outbreak During a Military Deployment in Central African Republic. Food Environ Virol 2017;9:498–501.

Webby RJ, Carville KS, Kirk MD, *et al.* Internationally distributed frozen oyster meat causing multiple outbreaks of norovirus infection in Australia. Clin Infect Dis 2007;44:1026–1031.

Westrell T, Dusch V, Ethelberg S, *et al.* Norovirus outbreaks linked to oyster consumption in the United Kingdom, Norway, France, Sweden and Denmark, 2010. Euro Surveill 2010;15:25.

Yu J-H, Kim N-Y, Koh Y-J, Lee H-J. Epidemiology of foodborne Norovirus outbreak in Incheon, Korea. J Korean Med Sci 2010;25:1128–1133.

Zomer TP, De Jong B, Kuhlmann-Berenzon S, *et al.* A foodborne norovirus outbreak at a manufacturing company. Epidemiol Infect 2010;138:501–506.
